# Discoveries Interview: Chad Walkaden cancer survivor on how to live a healthier and longer life while diagnosed with cancer

**DOI:** 10.15190/d.2019.1

**Published:** 2019-03-20

**Keywords:** The Cancer Blueprint, cancer survivor.

**Figure 1:**
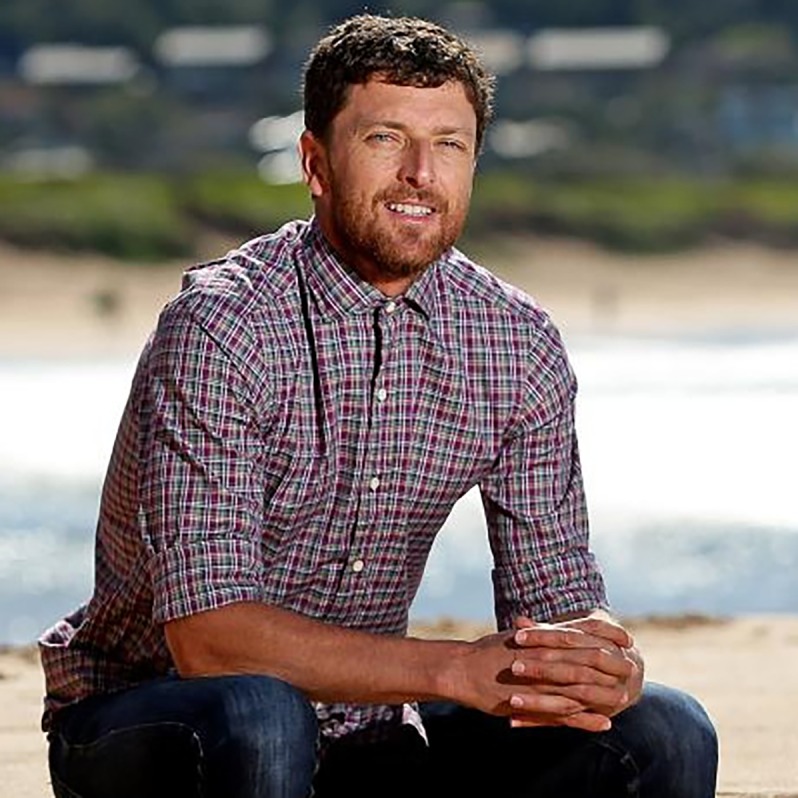
Chad Walkaden, a cancer survivor and the founder of The Cancer Blueprint.

Chad Walkaden is the Founder of The Cancer Blueprint^1^, Australia, a program developed to assist people to live better and longer lives. He is a qualified forensic social worker with over 17+ years of experience in working across the area of mental health in both private practice and for government in Australia and the United Kingdom.

His unique understanding of the struggles faced by individuals with emotional and psychological challenges results from his extensive professional experiences combined with his achievements of overcoming a terminal Stage IV Adrenal Cortical Carcinoma on three separate occasions.

Chad's work has been featured in several journals and media portals, including the Journal of Gastroenterology, Hepatology & Digestive Disorders and he presented at the International Conference on Gastrointestinal Cancer and Therapeutics, 4th World Congress on Digestive & Metabolic Diseases and 26th Annual Congress on Cancer Science and Targeted Therapies (2018)^2-4^.

## **1. Can you tell us about your experience with cancer?**

My experiences with cancer have encompassed a complete range of human emotions that were triggered when I was 29 years old and diagnosed with a terminal stage IV Adrenal Cortical Carcinoma (ACC).

This life changing terminal diagnosis signaled an unparalleled shift in my consciousness that is entirely the result of an existential fear about my death. My fear of death was not a singular thought. But, rather, a reoccurring, disturbing yet all capturing focus that was compounded by the array of changes, losses, worries and pain that are all still very easily to locate. Despite the presence of these harder and more difficult emotions, there was also an emergence of resilience, strength and an unwavering system of belief that was based around my future life. These latter factors proved to be of more importance particularly during the consecutive reoccurrences that I experienced while also simultaneously experiencing my mother face and overcome a stage 3 bowel cancer.

To this day, I see my experiences with cancer as directly linking to a wider conversation about mental health. But, maybe not the typical conversation that occurs. For me, tackling the myriad range of complex emotions was no easy task but it was crucial that I was able to address and then move beyond these vulnerabilities. Failure to do this would not have permitted me to maintain the level of physical, emotional and psychological well-being that is required to endure almost half a decade of continuous cancer treatment, multiple surgeries and the countless changes that occurred in my life.

## **2. How did you find the strength to go through the treatments?**

My strength was the result visible to others. At the core of this was a mindset that was continually constructed in a fashion not to indifferent to a child working tirelessly away at a 1000-piece jigsaw puzzle.

My mindset was based on two key beliefs. An initial belief was that I needed the discipline, training routines and psychology comparable to an Olympic athlete. I knew that I had to be stronger in my body, in my mind and in my spirit. I knew that every day mattered. That, I was competing in my own Olympics. But, instead of a podium finish. My gold medal was my life.

One of my other key components was the purpose and opportunity seen in my circumstances. At the time of diagnosis, I had completed seven years at university studying education, social work and family therapy. Additionally, I had over 10 years working in mental health across a range of roles in Australia and United Kingdom. In many ways, I was in a unique position that allowed me to draw on theory and research to assist me to turn my pain, losses and daily battles with chemotherapy into something greater than an individual fighting for his life. I needed to believe that my circumstances would matter for something greater than my own existence. That the daily side-effects of two years of straight chemotherapy would eventually improve the way that other people with cancer could overcome their physical, emotional, psychological and social difficulties.

## **3. Can you tell us about your amazing program that you have developed called The Cancer Blueprint and other ongoing initiatives that you have ongoing?**

At the core of The Cancer Blueprint is a genuine authenticity to assist people to live better and longer lives.

The multitude of my personal experiences brought to surface unknown vulnerabilities and existential fears that forced me expand on my pre-existing knowledge about human behavior and the ways that we respond to stressors, traumas and life challenges. This self-imposed mission to expand on my learning led to me developing a methodology that combines the emotional, psychological and social factors that are associated with having cancer with an integrative approach that uses nutrition, movement, forms of meditation and other therapies to optimize the capacity of a patient to be in the best position possible to respond to the treatment provided by an oncologist.

At the time of writing, The Cancer Blueprint has passed three stages of early testing and is in need of more rigorous testing. The most recent evaluation of this program was completed in 2018 when 100 participants were introduced to a digital version of my methodology. In the pilot, participants received bite-sized digital information directly to their phone and/or computer, in a format designed to cater to the likely various learning styles of the participants. The results were again promising and indicated the potential of a digital version of The Cancer Blueprint to play a key role in reducing isolation for patients, supporting patients to reduce anxiety and also improving the capacity for patients to cope.

## **4. How do you see this field evolving in the next 5-10 years?**

Throughout early stage testing, our results suggest that patients could greatly benefit from a digital solution that assists them to better manage the physical, emotional, psychological, and social complications that are known to place them at higher risk of developing a range of complex health needs that impact their mental health and quality of life.

In the USA alone, the number of people diagnosed with cancer is an estimated 1,735,350. Added to this number is the estimated 15.5 million cancer survivors. By 2026, this figure is expected to rise to 20.3 million people. This alarming figure highlights the need for a cost-effective digital solution that can promote accessibility of proven strategies and support to patients across geographical locations that are from a variety of cultural and economic backgrounds.

## **5. What three things would you tell a person who just yesterday learned of their cancer diagnosis and to everyone diagnosed with cancer?**

My first response would not be to say anything. Instead, I would want to curiously listen to their words, observe their body language and watch for the changes in their behavior/language when they speak so that I could learn about their pre-existing strengths and vulnerabilities.

To everyone else with cancer, I would encourage them to:

**A**. Recognize there are going to be down days and that it is normal to have feelings of sadness, anxiety and fear.

**B**. Consider renewing an interest or starting a new hobby that can directly be associated to either cognitive or physical benefits. Preferably, this would be undertaken with a friend, family member or community and be a focus at least three times per week.

**C**. Think of their body as a business. Recruit the best team of people possible to give the best chances of living long and living well.

**D**. Adopt all the 10 strategies from The Cancer Blueprint into their lives. Honestly, it is the best advice I have.
